# Health Benefits and Chemical Composition of Matcha Green Tea: A Review

**DOI:** 10.3390/molecules26010085

**Published:** 2020-12-27

**Authors:** Joanna Kochman, Karolina Jakubczyk, Justyna Antoniewicz, Honorata Mruk, Katarzyna Janda

**Affiliations:** Department of Human Nutrition and Metabolomics, Pomeranian Medical University, 24 Broniewskiego Street, 71-460 Szczecin, Poland; kochmaan@gmail.com (J.K.); kaldunskajustyna@gmail.com (J.A.); honoratamruk15@gmail.com (H.M.); Katarzyna.Janda@pum.edu.pl (K.J.)

**Keywords:** matcha, green tea, catechins, EGCG, *Camellia sinensis*, chemical composition, health-promoting effect, polyphenols

## Abstract

Japanese matcha is a type of powdered green tea, grown in a traditional way. Shading of the plants during the growth period enhances the processes of synthesis and accumulation of biologically active compounds, including theanine, caffeine, chlorophyll and various types of catechins. Green tea contains four main catechins, i.e., (−)-epicatechin (EC), (−)-epicatechin-3-gallate (ECG), (−)-epigallocatechin (EGC) and (−)-epigallocatechin-3-gallate (EGCG), of which the latter is the most active and abundant and matcha is their best condensed source. Due to its unique chemical composition and prized flavour, which sets it apart from other tea beverages, it is considered the highest quality tea. Its health-promoting properties are attributed to the high content of antioxidant and anti-inflammatory substances. Studies confirming the high antioxidant potential of tea beverages claim that it originates from the considerable content of catechins, a type of phenolic compound with beneficial effects on human health. Due to its potential for preventing many diseases and supporting cognitive function, regular consumption of matcha may have a positive effect on both physical and mental health. The aim of this review was to compile the health benefits of matcha tea. It is the first such review to be undertaken, and presents its main bioactive compounds in a systematic manner.

## 1. Introduction

Tea is one of the most consumed beverages, second only to water, in many societies [1]. Its distinctive flavour, aroma and health-promoting effects are highly valued around the world, as are its socio-cultural connotations [2,3]. Green tea is available in many variants: in the form of loose leaves, packed into teabags or powdered [4]. Matcha is a powdered type of Japanese green tea (*Camellia sinensis)* of the Tencha variety [5]. The popular beverage has been growing around the world [6]. It is particularly rich in antioxidant compounds as a result of the special cultivation method [7,8]. According to the traditional method, for the majority of the growth period, the tea bushes are covered using bamboo mats to shade the leaves from excessive direct sunlight [4]. In the course of this process, plants are able to produce higher amounts of amino acids and bioactive compounds, including chlorophyll and theanine, responsible for the unique, non-bitter taste and the characteristic, vibrant colour of matcha. As a result, matcha is highly valued for its quality and regarded as the most aromatic green tea [5,9].

## 2. Chemical Composition of Japanese Matcha Green Tea

The health benefits of green tea arise from the presence of natural antioxidants [10], such as polyphenols: a wide range of compounds accounting for as much as 30% of the dry weight of green tea [2,11]. Polyphenols are believed to be exceptionally powerful antioxidants, with effects comparable to those of vitamins, such as vitamins C and E, carotene and tocopherol [12,13,14]. The amounts of health-promoting active substances contained in tea beverages depend of the type of tea, the amount of tea leaves per portion, brewing temperature and time [15].

### 2.1. Content of Catechins

Studies confirming the high antioxidant potential of tea beverages claim that it originates from the considerable content of catechins, a type of phenolic compound with beneficial effects on human health [1,10,16,17,18,19,20,21]. Green tea contains four main catechins, i.e., (−)-epicatechin (EC), (−)-epicatechin-3-gallate (ECG), (−)-epigallocatechin (EGC) and (−)-epigallocatechin-3-gallate (EGCG), of which the latter is the most active and abundant [22,23]. High polyphenolic content has a greater capacity for scavenging free radicals than vitamin C on its own. Phenolic compounds occur naturally in the leaves of *Camellia sinensis*. Matcha may therefore be described as a major source of catechins in daily human diet [1,10,16,17].

Epigallocatechin gallate (EGCG), epigallocatechin (EGC), epicatechin gallate (ECG) and epicatechin are the main active compounds of the catechin type and are therefore present in the highest amounts in plant products [24]. Catechins derived from tea demonstrate outstanding antioxidant activity due to their ability to neutralise free radicals and boost the detoxification activity of enzymes, including glutathione peroxidase, catalase and glutathione reductase [8,25]. Grzesik et al. [26] reported that catechins have greater antioxidant capacity than glutathione, vitamin C and flavonoids, which attests to their key role in maintaining cellular redox homeostasis.

According to the study by Koláčková et al. [27], the total polyphenolic content found in matcha tea falls within the range of 169–273 mg GAE/g. However, Nishitani and Sagesaka [28] observed a lower polyphenol content in matcha than in other green teas. It was then suggested that such a result may be due to the shading of the tea plants, which inhibits polyphenol synthesis. Nevertheless, catechin content in green teas is much higher than in black teas, amounting to 5.46–7.44 mg/g, compared with 0–3.47 mg/g in black tea [29].

### 2.2. Content of Caffeine

Caffeine is an essential component of tea beverages and is responsible for their distinctive and desirable taste. At the same time, it is a powerful antioxidant adding to the antioxidant potential of the beverage [27]. Its level may be associated with the time of harvest and age of leaves—the older the leaves, the lower the caffeine content. Caffeine content also depends on tea variety, weather conditions during vegetation, as well as the brewing method [27]. The effects of caffeine are rooted in its antioxidant potential, neutralising reactive oxygen species and enhancing antioxidant enzyme activity and total glutathione levels. In regular doses, caffeine may reduce persistent oxidative stress, bringing down the prevalence of free radical-mediated diseases [30]. Additionally, caffeine may inhibit the secretion of proinflammatory cytokines, demonstrating anti-inflammatory effects [31].

Matcha has a relatively high caffeine content compared to other green teas, which gives it a unique aroma and flavour [29]. The content of caffeine in green teas was found to fall within the range of 11.3–24.67 mg/g [32], while in matcha it amounted to between 18.9 and 44.4 mg/g [27]. For the sake of comparison, most coffee beans will contain 10.0–12.0 mg caffeine/g of beans [27].

### 2.3. Content of Phenolic Acids

Phenolic acids are secondary plant metabolites, characterised by high antioxidant and anti-inflammatory potential, in addition to neuroprotective and hypoglycemic effects [30,33]. They have also been reported to inhibit cancer cell growth and prevent metastasis [34]. Some phenolic acids, through modulating lipid and carbohydrate metabolism, may support the regulation of metabolic disorders [35]. One of the most common compounds from this group found in foodstuffs is chlorogenic acid [27,35].

In a study by Koláčková et al. [27], the total content of phenolics in alcoholic extracts was determined to reach up to 273 mg GAE/g. Detailed analysis revealed the following maximum levels of phenolic acids in matcha tea samples, differing in terms of various criteria, including origin: gallic acid—423 μg/g, p-hydroxybenzoic acid—243 μg/g, chlorogenic acid—4800 μg/g, caffeic acid—223 μg/g, ferulic acid—289 μg/g and ellagic acid—371 μg/g [27].

### 2.4. Content of Rutin

Rutin, which is a polyphenolic compound, is a potent antioxidant. Its synergistic interaction with ascorbic acid may enhance the protective effects of both substances in the cardiovascular system, strengthening blood vessels [36]. It also has antidiabetic and anti-inflammatory properties, thus preventing diabetes-related pathologies [37,38]. Its antioxidant and anti-inflammatory action offers potential for preventing conditions of free-radical or inflammatory origin, including neurodegenerative conditions [39].

According to Jakubczyk et al., [36] matcha green tea has an exceptionally high rutin content, compared to other teas available in the market. The authors contrasted the level of rutin found in matcha (1968.8 mg/L) with that in buckwheat (62.30 mg/100 g), the latter being recognised as one of the richest sources of rutin in the human diet, and demonstrated that matcha tea may be a better source of the compound than other foodstuffs. The findings made by Jakubczyk et al. [36] appear to be consistent with the observations of Koláčková et al. [27].

### 2.5. Content of Quercetin

Quercetin is a phytochemical with antioxidant and neuroprotective activity [40,41,42]. Additionally, it was observed to normalize carbohydrate metabolism by inhibiting glucose absorption from the gastrointestinal tract, regulating insulin secretion and improving insulin sensitivity in tissues [43]. What is more, the combination of quercetin with (−)-epigallocatechin gallate (EGCG) may enhance the anticarcinogenic effects of both [6].

The content of quercetin in the aqueous extract of matcha was measured by Schröder et al. [6] at 1.2 mg/mL, which is marginally higher than in traditional green tea (1.1 mg/mL). On the other hand, Koláčková et al. [27] determined quercetin levels in alcoholic extracts reaching up to 17.2 μg/g.

### 2.6. Content of Vitamin C

Vitamin C is a powerful exogenous antioxidant. Due to its properties, it reinforces the immune defence of the body. It is an essential micronutrient in human nutrition which should be supplied every day in adequate amounts [44,45].

Jakubczyk et al. [36] demonstrated that infusions of matcha tea contain from 32.12 to 44.8 mg/L of vitamin C, depending on the temperature of water used to prepare the infusion and the type of tea. In the study by Koláčková et al. [27], matcha was found to contain more than double the amount of vitamin C of other green teas. Its content was determined at 1.63–3.98 mg/g, depending on the type of product and its origin.

### 2.7. Content of Chlorophyll

Thanks to shade-growing, matcha tea has increased chlorophyll content, which is responsible for its unique vibrant colour [46]. Chlorophyll and its derivatives exhibit strong antioxidant and anti-inflammatory activity [47].

The levels of bioactive compounds, including chlorophyll, in the Tencha-type tea leaves which are used specifically for matcha were determined by Ku et al. [48]. The level of chlorophyll in Tencha leaves was higher than in traditional green tea, amounting to 5.65 mg/g and 4.33 mg/g, respectively.

### 2.8. Content of Theanine

Theanine is an amino acid found in the tea plant *Camellia sinensis*. Due to the shade-growing of plants intended for matcha production, theanine does not break down. As a result, Tencha leaves contain larger amounts of it compared to other teas [49]. The relatively high theanine content in matcha tea is responsible for its unique non- bitter taste, and in combination with caffeine provides the taste sensation and umami characteristic of this type of tea [48,50]. The combination of l-theanine and caffeine may enhance concentration, vigilance and efficiency to a higher extent than the use of either compound alone [51], additionally alleviating stress [49].

According to Kaneko et al., the content of l-theanine in matcha tea infusions amounts to 6.1 mg/L [50], while Unno et al. [9] found as much as 44.65 mg/g of that compound in matcha tea samples.

**Figure 1:**
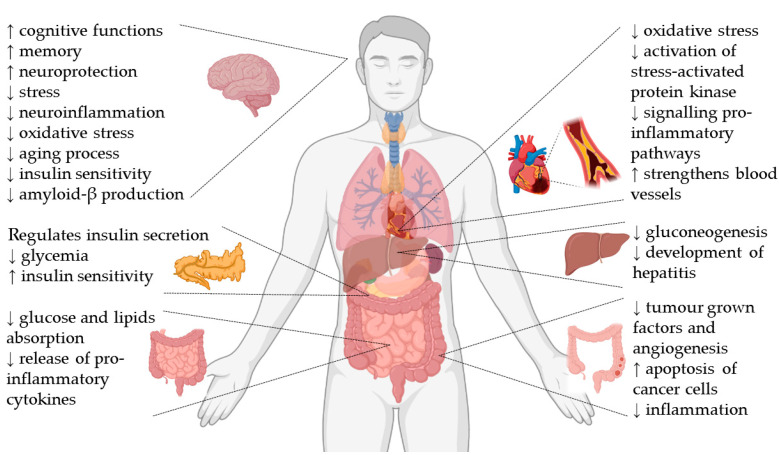
Health-promoting properties of the main bioactive compounds of matcha green tea.

A summary of the health-promoting properties of the main bioactive compounds of matcha green tea is presented in Figure 1.

## 3. Parameters Affecting Chemical Composition

One parameter which has a significant effect on the chemical composition and health-promoting properties of a tea beverage is the temperature of the water used to make the infusion. This is related to the extraction of biologically active compounds and higher kinetic energy in tea brewed at a high temperature [2,36,52].

The distinctly high antioxidant potential of matcha can also be attributed to the grinding process and, ultimately, the powdered form. Fujioka et al. [52] demonstrated that infusions made by steeping tea leaves have a lower polyphenol content than those made from the powdered form. Thus, the grinding process itself may accelerate the extraction of polyphenolic compounds. Shishikura and Khokhar [53] observed, taking into account the average time it takes to prepare tea, that its powdered version is more effective and active in terms of the extraction in a relatively shorter time (1 min), and therefore seems to be a better choice. Komes et al. [2] examined 11 green teas differing in terms of the manufacturing process and form, including bagged, loose leaf and powdered tea; matcha brewed at three temperatures—60, 80 and 100 °C; and over different durations, i.e., 3, 5, 10, 15 and 30 min. The scholars measured the impact of leaf fragmentation, form of product, brewing time and temperature on phenolic content and antioxidant capacity, determined using different methods (DPPH, FRAP and ABTS). In all green teas, antioxidant capacity increased together with the temperature of water used to prepare the infusion, and the optimal values were observed at the highest temperature with a 3-min brewing time. It was concluded that the antioxidant potential of green tea increases proportionately to its phenolic content. Additionally, the powdered form had the highest parameters of all the green teas, and the required extraction time was the shortest. Extending the brewing time of powdered matcha did not increase its antioxidant capacity [4].

The content of health-promoting substances, including polyphenols, is also affected by the agro-climatic conditions during growth, such as the number and distribution of sunny and rainy days, fertilisation and plant protection measures, if any, etc. [54].

## 4. Health-Promoting Properties

### 4.1. Anticarcinogenic Effects

The anticarcinogenic properties of green tea and its key ingredient, (−)-epigallocatechin gallate (EGCG), have been thoroughly researched by scholars from around the globe [40,55,56,57,58,59]. The mechanisms behind the anti-cancer effect of EGCG may be related to inhibiting tumour angiogenesis, antioxidant effects and suppressing the inflammatory processes contributing to transformation, hyperproliferation and initiation of carcinogenesis [57,59].

The pathogenesis and progression of colorectal cancer is significantly affected by healthy diet and lifestyle. Obesity, especially of the visceral type, as a consequence of long-standing unhealthy lifestyle choices, increases the risk of developing gastrointestinal cancer [60]. Consuming large amounts of EGCG may contribute to reducing the incidence of colorectal cancer, partly due to inhibiting tumour growth factors. What is more, EGCG is capable of inhibiting growth and inducing apoptosis of cancer cells [58]. Improving tissue sensitivity to insulin and leptin, and reducing blood lipid parameters, may inhibit obesity-related carcinogenesis. Supplementation of green tea extracts may additionally prevent recurring adenomas, which in the majority of cases may evolve into colorectal cancers [58]. Research findings regarding EGCG supplementation also include inhibition of growth and proliferation of gallbladder and bile duct cancer cells, as well as a decreased risk of biliary duct cancer [40,57].

Catechins act synergistically with anticancer medications, and can be used to support therapy as well as in cancer prevention [61]. Vitamin C has also been associated with protective effects against cancer [62].

### 4.2. Anti-Inflammatory Effects

Inflammatory response is part and parcel of many diseases. It may lead to the production of excessive amounts of substances promoting the production of reactive oxygen species (ROS), which can damage cell structures and lead to long-term disruption in the functioning of the body as a whole, as well as playing signalling functions promoting inflammation. The main effect of anti-inflammatory and antioxidant substances is to inhibit signalling in the inflammatory process by scavenging ROS [63].

Supplementation of EGCG, the main bioactive component of green tea, may alleviate complications of the inflammatory process arising after the use of cardiopulmonary bypass for major cardiac surgery, including lung injury and dysfunction [64,65]. By regulating the inflammatory condition, EGCG also helps reduce the susceptibility to gallstone formation. Arterial hypertension is controlled by multiple genes, with inflammation and vascular remodelling implicated in the pathogenesis of this condition [66,67]. Consumption of green tea beverages with a high content of bioactive compounds regulating inflammatory processes attenuates the development of hepatitis, by suppressing gene and protein expression of inflammatory cytokines [22].

### 4.3. Cardioprotective Effects

Cigarette smoking is recognised as one of the main risk factors for cardiovascular diseases [68]. In an experiment with an animal model, rats were exposed to cigarette smoke, with simultaneous oral administration of EGCG [69]. Upon exposure to smoking, the markers of myocardial injury and lipid anomalies were elevated. Administration of EGCG reversed these aberrations. The findings from that study suggest that the antioxidant EGCG may exert a protective effect on the heart muscle by preventing cardiac inflammatory changes via reducing oxidative stress [69]. EGCG may potentially exert a protective effect on the heart muscle in patients undergoing surgery who are susceptible to ischemic injury, by inhibiting the activation of stress-activated protein kinase and signalling pathways inducing the inflammatory response [70,71].

### 4.4. Antiviral Properties

The immunomodulatory properties of green tea and its antiviral effect may support the prevention and regulate immune response in infectious diseases, including COVID-19 [72,73]. There are many studies on the antiviral properties of green tea, however they are mostly based on reports about traditional green tea [74,75,76,77,78]. The mechanism of action and specific properties of matcha green tea are still unknown and equated with general reports on green tea, despite the different composition and ratio of bioactive compounds [2,4,36,56]. However, in one of the few studies, Ohgitani et al. [79] demonstrated that matcha green tea may have antiviral activity (by inactivating SARS-CoV-2), which is a promising report, but requires more detailed research. Documented potential properties and probable mechanism of action of green tea compounds are presented in Table 1.

### 4.5. Potential for Regulating Carbohydrate Metabolism

The effects of catechins and other polyphenolics on the parameters of carbohydrate metabolism show their hypoglycemic action [89]. Matcha may help lower blood glucose levels [90], and its EGCG content may inhibit starch digestion, thus minimising the sudden release of glucose in the gastrointestinal tract [91]. EGCG may present capacity for inhibiting gluconeogenesis and the absorption of lipids and glucose from the gastrointestinal tract, as well as improving insulin sensitivity [89].

### 4.6. Improvement of Cognitive Function, Prevention of Neurodegenerative Disorders

Consumption of green tea is regarded as an effective dietary intervention to promote clarity of mind and cognitive function. These health benefits are attributed mainly to epigallocatechin gallate (EGCG) [92].

Cognitive function tends to deteriorate with age in a manner dependant on environmental factors, including lifestyle [93]. Regular dietary intake of caffeine may reduce the risk of cognitive decline in women, and its effect increases with age [94]. By reversing oxidative processes and reducing neuroinflammation, caffeine may indirectly inhibit ageing of the brain [95], and in this way maintain its normal function. Oxidative stress, which is capable of inducing neuronal damage, may induce memory impairment. Caffeine supplementation, with its anti-inflammatory effects, chiefly in the hippocampus, may prevent the development of this disorder [96]. The positive effects of caffeine on the nervous system and preventing neurodegenerative diseases are closely related to the decreased deposition of amyloid-β in the brain [97]. Systemic inflammation induced by lipopolysaccharide (LPS) plays a key role in neurodegenerative diseases. EGCG inhibits LPS-induced production of reactive oxygen species, suggesting that EGCG is a potent and effective neuroprotective agent in neurological disorders mediated by inflammation [98].

EGCG intake enhances cognitive function, improves insulin sensitivity and decreases amyloid-β production in the brain, thus reducing neuroinflammation and preventing neuropathologies related to neurodegenerative diseases, including Alzheimer’s disease [99].

### 4.7. Prospects

Matcha green tea, due to its unique composition of bioactive compounds, offers a wide range of potential health benefits, as presented in Table 2. It contains high concentrations of phenolic acids, quercetin, rutin, theanine and chlorophyll, exceeding those in other green tea varieties. It is a relatively new and unknown product, which cannot be identified with traditional green tea; it is a separate tea variety with distinct properties. Its infusions and extracts may find potential applications in preventing lifestyle diseases of free-radical and inflammatory origin, as well as in preventing premature ageing processes. Matcha tea, thanks to its powdered form, makes for an easy-to-use food additive. Unfortunately, the direct impact and mechanisms responsible for the properties of matcha tea have not been sufficiently explored. Hence, many of the potential aspects of its activity, e.g., its interactions with intestinal microflora and impact on infectious diseases, requires further study. It seems necessary to carry out more extensive research, including a detailed examination of the chemical composition of matcha tea, studies with the use of cell lines and animal models, and randomised clinical trials (RCT) to confirm the hypothesised beneficial effects of matcha tea on human health.

## 5. Conclusions

The Japanese powdered green tea, matcha, contains high amounts of substances with antioxidant and anti-inflammatory effects. It has promising potential health benefits, mainly through a high concentration of catechins. With regular consumption, it may support the body’s efforts to maintain health and prevent disease. Research into the effects of matcha drinking and its individual components in specific disease entities is still valid and needed. The current state of knowledge only covers some of the health-promoting properties of this tea. To confirm the validity of implementing recommendations for increased consumption of tea beverages made from matcha, it will be necessary to undertake deeper and broader analyses of its effects on the human body.

## Figures and Tables

**Table 1:** Research on antiviral properties of green tea compounds.

Compound Related to the Effect	Potential Mechanism and Properties	References
EGCG	Antiviral effect depends on virus type e.g. inhibiting replication of HIV-1, inhibiting viral (HBV) entry to the cell, inhibiting first stages of infection, inactivate SARS-CoV-2, inhibiting SARS-Cov-2 main protease and SARS-CoV-2 3C-like protease, binding to viral surface proteins	[78], [80], [77], [81], [79], [82], [83], [84]
Catechins	Inhibiting adherention and cell penetration, disruption of the viral replication cycle, inhibiting HCV replication, anti-inflammatory, inhibiting SARS-Cov-2 main protease	[85], [76], [86], [87]
Quercetin	Inhibiting SARS-Cov replication by inhibition of SARS-Cov-3C-like protease	[88]
Catechins, quercetin	Inhibiting COVID-19 main protease and structural proteins	[73]

**Table 2:** Summary of research on the health-promoting properties of matcha green tea.

Health-Promoting Properties	The Component Associated with the Effect	Mechanism of Action	Reference
Anticarcinogenic effects	Catechins	support therapy as well as in cancer prevention, inhibiting tumour growth factors and inducing apoptosis of cancer cells	[58], [61]
	Vitamin C	protective effects against cancer	[62]
	Phenolic acids	inhibiting cancer cell growth and prevent metastasis	[34]
	EGCG	inhibiting tumour angiogenesis, antioxidant effects and suppressing the inflammatory processes contributing to transformation, hyperproliferation and initiation of carcinogenesis, improving tissue sensitivity to insulin and leptin, and reducing blood lipid parameters;	[57], [58], [59], [61]
Anti-inflammatory effects	EGCG	scavenging ROS, regulating the inflammatory condition and response	[22], [66]
**Cardioprotective**	EGCG	reducing oxidative stress, inhibiting the activation of stress-activated protein kinase and signalling pathways inducing the inflammatory response	[69], [70]
	Rutin	strengthening blood vessels	[36]
**Improvement of cognitive function and prevention of neurodegenerative disorders**	EGCG	promote clarity of mind and cognitive function, inhibits LPS-induced production of reactive oxygen species, improves insulin sensitivity and decreases amyloid-β production in the brain	[92], [98], [99]
	Caffeine	reduce the risk of cognitive decline, reversing oxidative processes and reducing neuroinflammation, inhibit ageing of the brain, anti-inflammatory effects, decreased deposition of amyloid-β in the brain	[94], [95], [96], [97]
Regulation of carbohydrate metabolism	EGCG	inhibiting starch digestion, inhibiting gluconeogenesis and the absorption of lipids and glucose, improving insulin sensitivity	[89], [91]
	Quercetin	inhibiting glucose absorption, regulating insulin secretion, improving insulin sensitivity	[43]
	Phenolic acids	modulating lipid and carbohydrate metabolism	[35]
